# Fatal stray dog attack in Russian Federation: a case report based on CCTV documentation

**DOI:** 10.1007/s12024-025-01015-6

**Published:** 2025-05-10

**Authors:** Galina Zolotenkova, Rizky Merdietio Boedi, Oleg Viktorovich Lysenko, Nikolaos Angelakopoulos

**Affiliations:** 1 Department of Forensic Medicine, I.M. Sechenov First Moscow State Medical University Moscow, Moscow, Russian Federation; 2 Department of Dentistry, Faculty of Medicine, Universitas Diponegoro, Semarang, Indonesia; 3 Moscow Regional Research and Clinical Institute (MONIKI), Moscow, Russian Federation; 4 Department of Orthodontics and Dentofacial Orthopedics, University of Bern, Freiburgstrasse 7, Bern, 3010 Switzerland

**Keywords:** Case report, Dog fatal attack, Russian Federation, CCTV footage, Wounds and injuries, Postmortem examination

## Abstract

Fatal dog attacks, though relatively rare, remain a significant public safety concern, particularly in regions with large stray dog populations. This case report details a fatal stray dog attack on a 77-year-old man in the Greater Moscow region, as documented through forensic examination and CCTV footage. The autopsy revealed extensive bite-related injuries, including severe vascular damage that led to exsanguination. The footage provided a unique and detailed account of the attack’s sequence, highlighting the prolonged aggression exhibited by the dog. This case underscores the importance of forensic investigations, surveillance footage, and effective stray dog management strategies to mitigate similar incidents. Further research is needed to better understand the risk factors associated with fatal dog attacks and develop targeted prevention measures.

## Introduction

The total biomass of domestic dogs is equivalent to the combined biomass of all land-dwelling mammal species [1]. While domestic dogs are widely regarded as loyal companions, guides, therapeutic aids, and valuable allies to humans [2], interactions with dogs—particularly stray dogs—remain a global concern due to public safety issues and the potential for attacks [3]. Freely roaming owned, stray, and feral dogs can significantly affect animal populations in natural habitats. These dogs present multiple threats to wildlife, including competition [4], increased mortality, and predation [5].

Animal bites, a common cause of primary and emergency care for both adults and children, pose significant risks to human health. These injuries not only cause physical trauma but also carry a high potential for infection, further complicating treatment and contributing to morbidity and mortality [6]. Alarmingly, there has been a rising trend in fatal dog attacks across Europe in recent years [7]. It was estimated by Weber et al. (1991) that approximately 300 to 700 animal bite incidents occur annually per 100,000 population [8]. According to the World Health Organization (WHO), there are no global estimates for the incidence of dog bites. However, research indicates that dog bites result in tens of millions of injuries each year [9].

The Russian Federation, with its vast territory spanning 17.1 million square kilometers and a population of approximately 146 million, is home to diverse ecosystems that support a wide array of wildlife and domestic animals. Among these, stray dogs (*Canis lupus familiaris*) have emerged as a growing concern, particularly in urban and peri-urban areas [10, 11]. Unlike large carnivores such as bears and wolves, which are largely confined to wilderness regions and are the focus of extensive conservation efforts, stray dogs thrive in human-dominated environments. In some cases, stray dogs form packs and display behaviors reminiscent of their wild ancestors, such as territoriality and predatory instincts [12–14]. Vulnerable populations, including children [15–18] and the elderly [5, 19], are particularly at risk during such incidents.

Efforts to manage stray dogs in Russia have evolved significantly over time. Historically, humane irrevocable capture methods helped reduce dog-related injuries. For instance, until 2014, these methods were used alongside other measures, leading to a gradual decline in emergency room visits related to animal bites. However, with the banning of these practices and the exclusive reliance on a Trap/Neuter/Return (TNR) policy, known as the OSV (*Отлов-Стерилизация-Возврат*) program, dog attacks increased by 9.2% by 2015 [20]. Public fear and frustration with the growing stray dog population led citizens to resort to unauthorized and inhumane methods, such as poisoning, as widely reported in the media by 2017 [21].

Despite efforts such as the Moscow Government’s Decree on TNR policies in 2022 (No. 819-PP), these challenges persist, underlining the need for more robust and adaptive management strategies. In 2019, Russian media frequently reported on proposed legislation to introduce taxes on domestic animals, purportedly set to be enacted in 2020. According to sources, the primary aim of this initiative was to enhance the welfare of domestic animals nationwide. However, critics contended that such measures might have the opposite effect, potentially exacerbating the issue of stray animals in the country [22].

Fatal dog attacks, although alarming, remain underreported and understudied in the Russian Federation. Research from other regions highlights factors such as environmental conditions, human behaviors, and stray dog population dynamics as contributors to such incidents. Effective control measures, including sterilization programs, public awareness campaigns, and improved waste management systems, have been shown to mitigate risks in similar contexts. However, comprehensive data specific to the Russian Federation is limited, particularly regarding fatal encounters, with media content often serving as the primary source of information on such events [23–25].

This paper aims to address a critical gap in the understanding of fatal stray dog attacks in the Russian Federation by presenting a detailed case report of an incident in the Greater Moscow region, as documented by the Moscow Regional Research and Clinical Institute and the Department of Forensic Medicine at I.M. Sechenov First Moscow State Medical University. By examining the circumstances, forensic findings, and contributing factors surrounding this event, the study aims to advance knowledge on human-stray dog interactions in urban contexts.

### Case report

This case involves a fatal attack on a 77-year-old man in the city of S., Moscow region, in November 2021. The incident occurred near the entrance of a building, where a stray dog attacked a female caretaker. The details of the event were established through video footage from the building’s CCTV system, which, incidentally, recorded the attack on the man by a single dog.

At 15:19:10, the woman was seen attempting to defend herself by hitting a roaming stray dog with a broom. At 15:19:35, the man (victim) entered the scene and was immediately attacked by the dog, which bit his right forearm. At this point, the woman continued trying to fend off the dog with the broom. At 15:20:13, all individuals disappeared from the camera’s view.

At 15:21:45, the dog reappeared, dragging the man into the camera’s field of view while continuing to bite down on the man’s forearm. The man attempted to defend himself again, and at 15:21:52, the dog bit the man in the neck, after which both the dog and the man vanished from the camera’s sight.

At 15:22:39, the woman reappeared, attempting to strike the dog with the broom. During this time, the dog was still clamping onto the man’s hand. By 15:24:44, the dog continued to bite the man’s hand, and at 15:25:22, the man was seen lying on the ground, bloodied, while the dog continued to attack his head and body. Throughout this period, the woman repeatedly tried to defend the man by striking the dog with the broom, pouring water on it, and attempting to cover it with a blanket.

At approximately 15:30:00, the man lost consciousness whilst the dog continues to bite and tear at his body, while pulling off his clothing until 15:33:00. From 15:33:00 to 15:59:53, the dog intermittently appeared and disappeared, circling the man. The footage ended at 15:59:59.

### Autopsy findings

The data for this case report were retrospectively collected from medical case files and forensic reports obtained from the Moscow Regional Research and Clinical Institute and the Department of Forensic Medicine at I.M. Sechenov First Moscow State Medical University. The investigation protocol received approval from the local Ethics Committee (number 04–24, dated 21/01/2024). A medico-legal autopsy was conducted in November 2021, approximately 20 h post-mortem, following the discovery of the deceased victim.

In the Russian Federation, forensic investigations follow standardized procedures to determine the nature of injuries and their mechanisms. The forensic pathology department performs comprehensive examinations, including the study of the skull, bones, and other biological materials from the deceased. Additionally, clothing with blood traces is analyzed to understand the formation of these patterns.

**Fig. 1:**
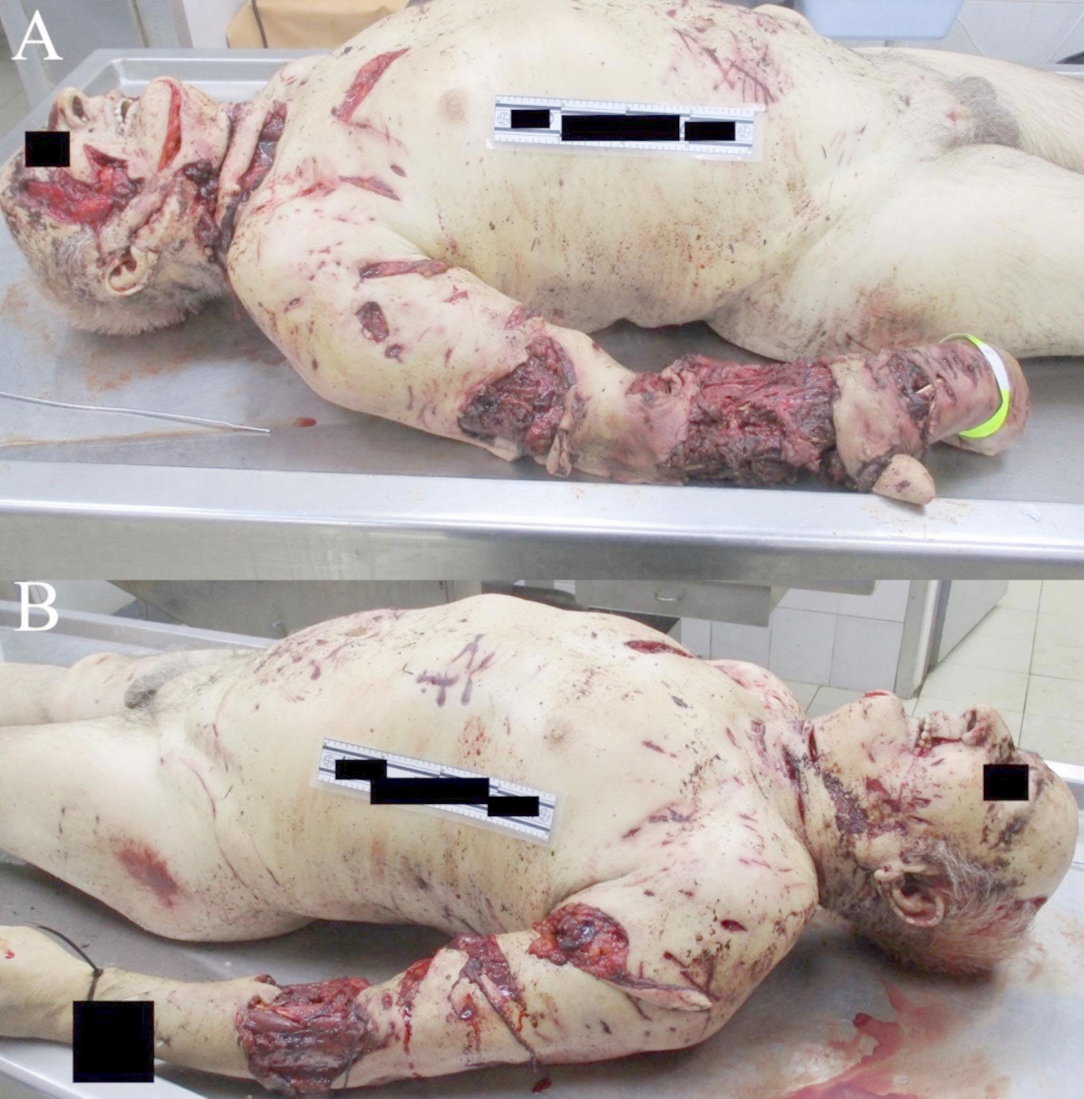
External examination of the victim with various type of wounds varying in size, length, and depth in both right (**A**) and left (**B**) side of the body

The victim presented with multiple through-and-through tears in his clothing across various surfaces of the body, characterized by frayed, uneven edges with pointed ends. Numerous lacerations and avulsions of varying depths and lengths were observed, consistent with sharp trauma, primarily concentrated on the right side (Fig. 1). The deceased’s identity was confirmed through visual identification by relatives.

**Fig. 2:**
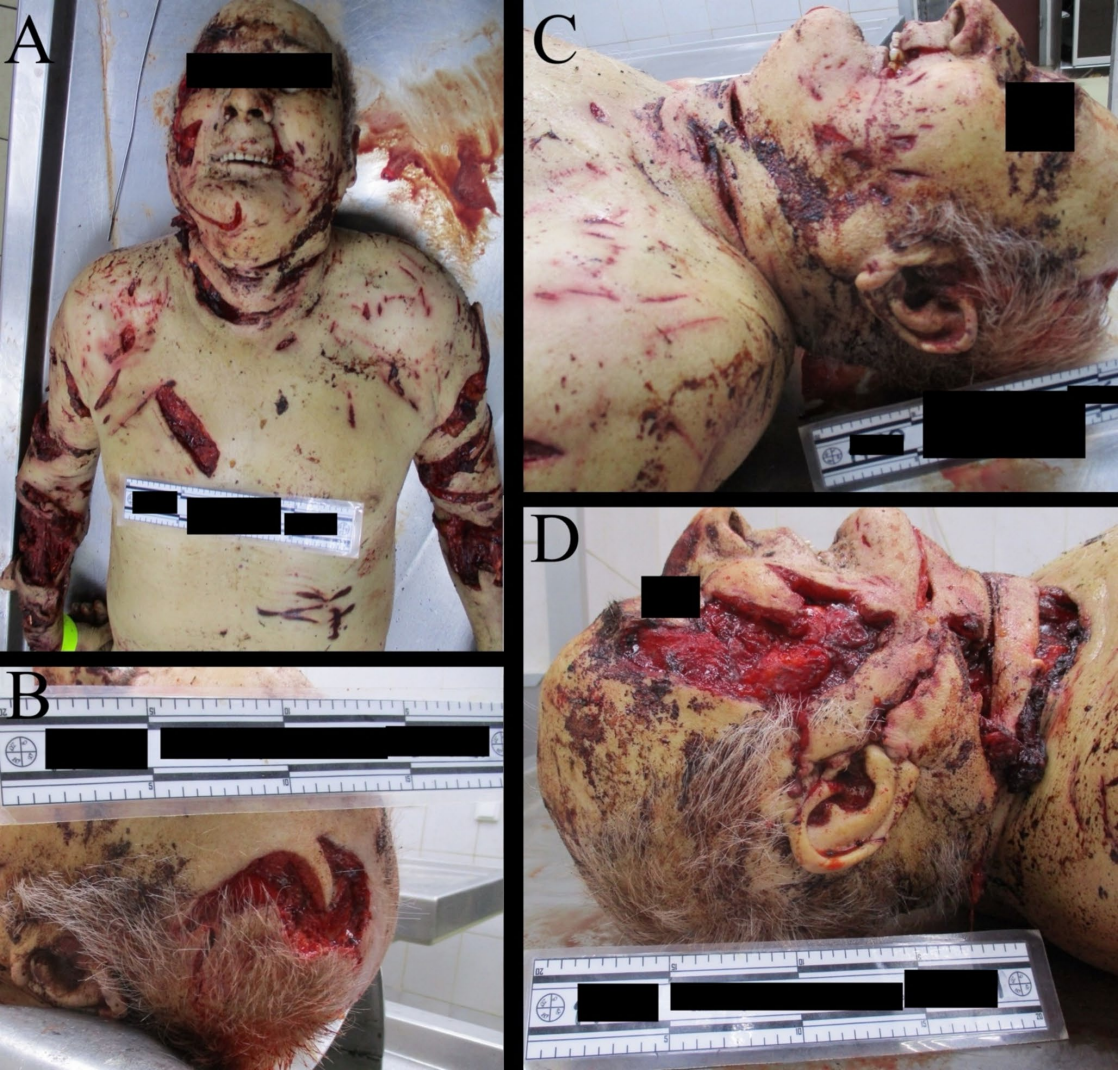
Overview of craniofacial injuries in the victim **(A)**, including the deep skin avulsion on the left parietal skull area **(B)** and zygoma area **(C)**. Deep lacerations were also observed in the sternocleidomasteoid region with severed external and internal jugular veins **(D)**

**Fig. 3:**
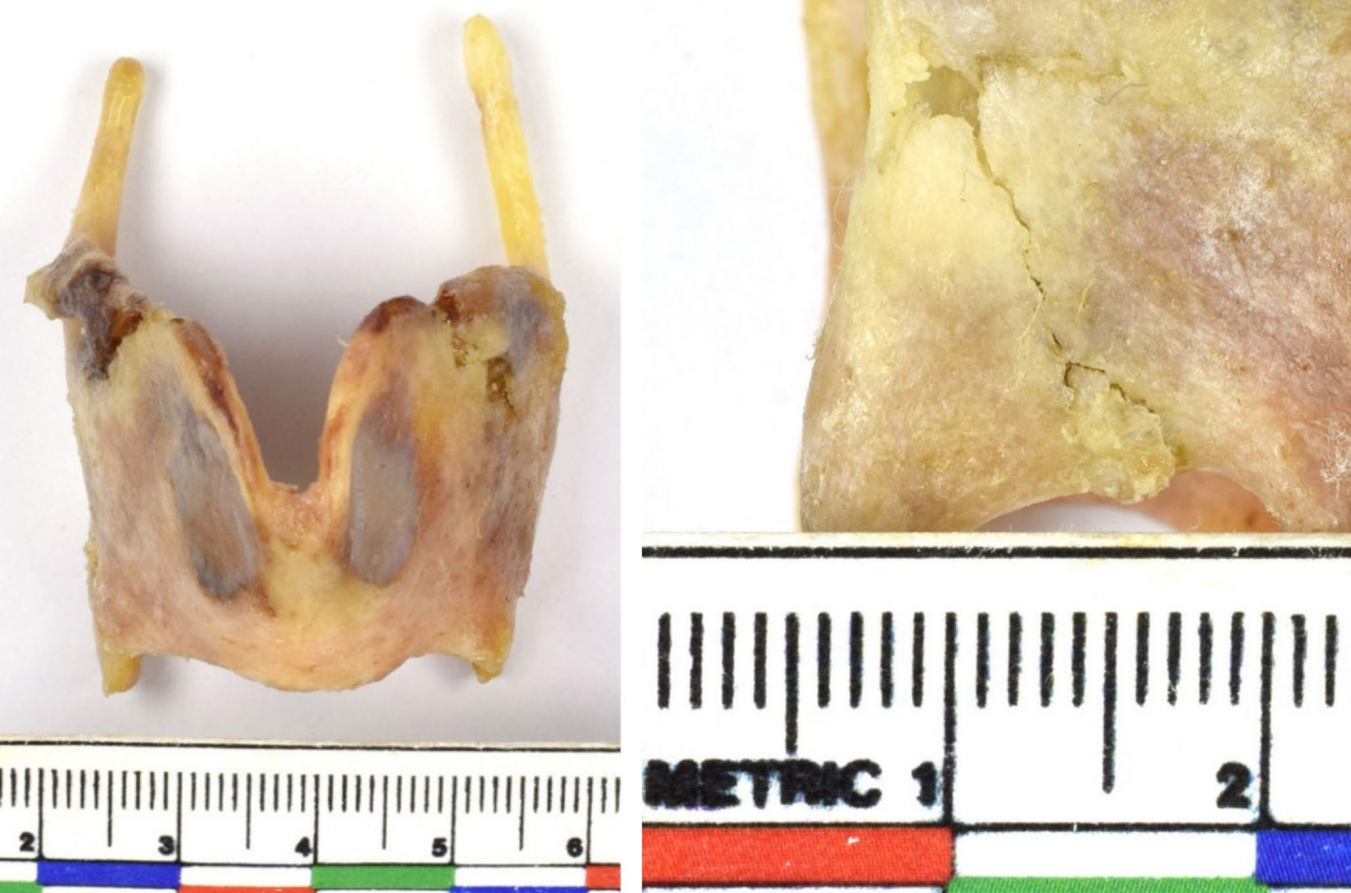
Damage to the right inferior horn of the thyroid bone due to trauma

Two primary wound types were identified: deep lacerations and puncture wounds consistent with canine clawing and biting. In the craniofacial region, multiple deep lacerations were noted, accompanied by extensive skin avulsions (Fig. 2). Severe trauma to both the right external and internal jugular veins was observed, likely resulting in significant hemorrhage and representing the principal cause of death (Fig. 2D). Additionally, damage to the right inferior horn of the thyroid cartilage was documented (Fig. 3).

**Fig. 4:**
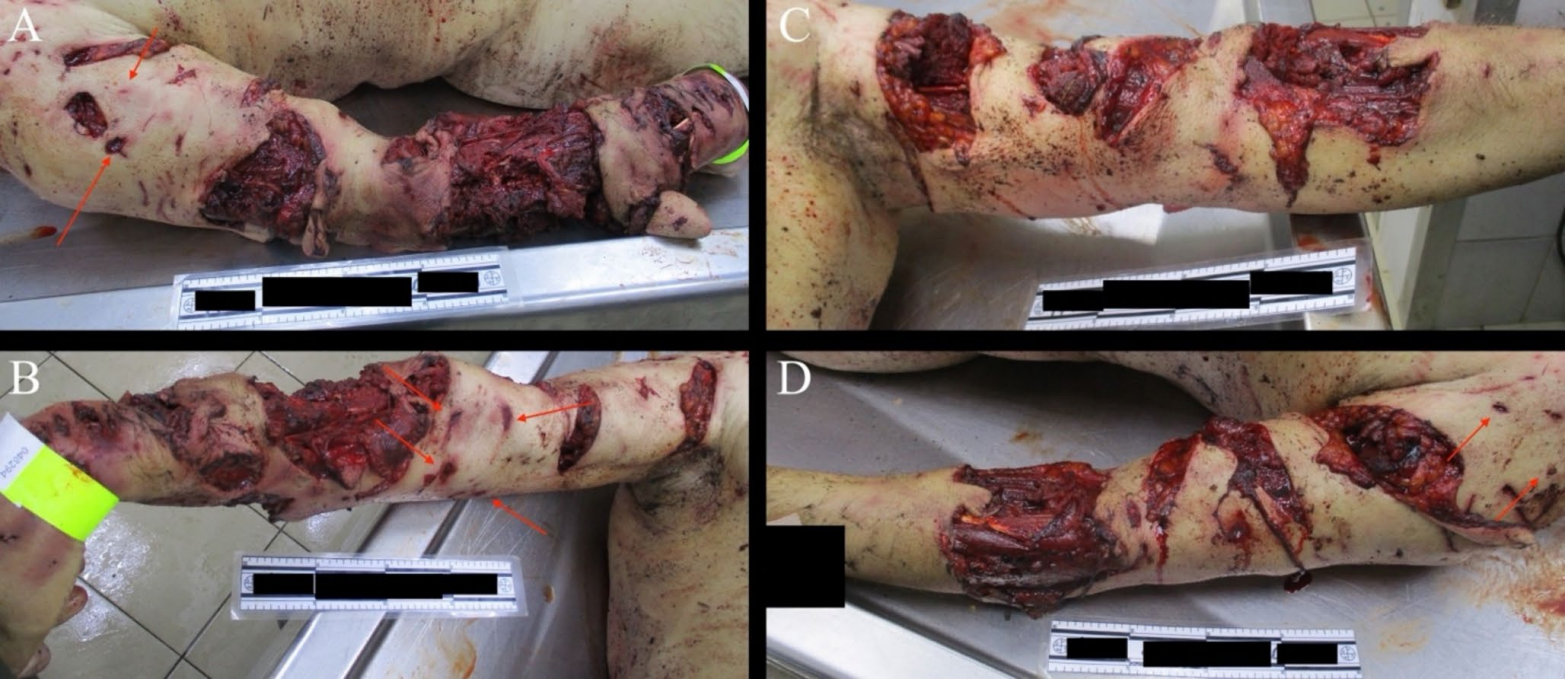
Severe lacerations and puncture wounds (red arrow) observed in both right (**A**, **B**) and left (**C**, **D**) hand

Multiple stab-like wounds were identified on both the left and right hands. These injuries were consistent with the inter-canine distance characteristic of bites inflicted by a carnivorous animal, accompanied by significant soft tissue damage and comminuted fractures of the left and right radius and ulna (Fig. 4). Analysis of CCTV footage confirmed that these wounds resulted from the dog’s claws and bites. However, due to the presence of avulsion injuries, it was not possible to reliably identify a definitive bite mark, as the full curvature and corresponding tooth pattern were not clearly imprinted on the wounds.

Further examination of the body revealed multiple abrasions and contusions, with several irregularly shaped bruises and abrasions observed on the lower extremities. On the anterior surface of the left shin, at the middle third, a cluster of abrasions and superficial wounds arranged in two arc-like formations was noted, with the convexity oriented laterally. The pattern and distribution of these abrasions were consistent with bite marks.

**Fig. 5:**
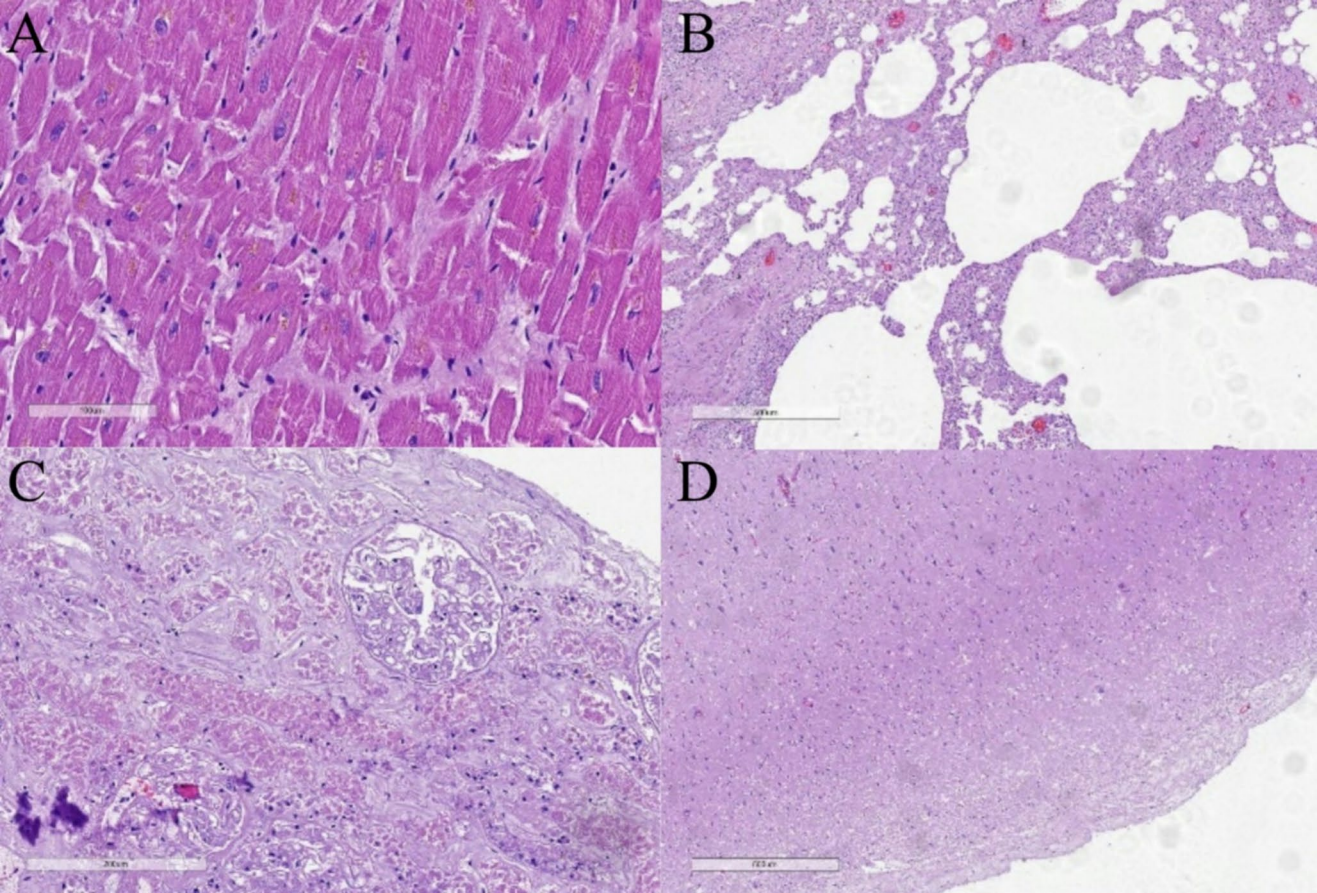
Histological findings on the autopsy. **A**. Myocardium, fragmentation of cardiomyocytes indication of acute blood loss, **B**. Lung, mild microcirculatory disorders wit collagenized alveoli and perivascular proliferation, **C**. Kidney, anemia of the medulla, **D**. Brain, sclerotic and edematous pia matter indicative of cerebral edema

The fatal injuries were attributed to multiple contused-lacerated wounds involving the soft tissues of the head, neck, and trunk. Extensive vascular trauma was also identified in the left brachial artery and vein, as well as in the subcutaneous veins of both upper limbs. The internal examination revealed severe anemia in the internal organs, indicative of significant blood loss prior to death, with histological examinations of various organs to confirm these findings (Fig. 5).

Taken together, these injuries are most consistent with claw and bite wounds inflicted by an animal, particularly a carnivore. While the severity and pattern of the injuries strongly suggest a dog attack, the identification of the animal was primarily supported by the CCTV footage. It is important to note that, in the absence of CCTV footage and eyewitness testimony, the distribution of these wounds alone would not have allowed for a definitive identification of the dog responsible and could have raised doubts regarding the specific cause of the trauma.

## Discussion

### Dog bites in Russian Federation and recent trends

In recent years, dog bites have become a significant public health concern in the Russian Federation. According to the Federal Service for Surveillance on Consumer Rights Protection and Human Wellbeing, approximately 238,000 individuals sought medical attention for dog bites in 2023, reflecting a 6.26% increase from 2020. In 2022, over 227,000 cases were recorded (155.35 per 100,000 population), accounting for 68.8% of all documented animal bite incidents—a proportion similar to 2021, which reported 68.5% of cases with over 228,000 medical visits.

Dog attacks predominantly occur in industrial zones, garages, and railway transport facilities (43%), followed by private sectors and dachas (33%). Stray or free-ranging dogs were implicated in 20% of cases; however, in private sectors, the true proportion of fatalities involving free-roaming dogs may be as high as 33% [26, 27]. While fatalities from dog attacks remain rare, non-fatal injuries are the most common outcome.

The COVID-19 pandemic influenced dog bite trends. A sharp decline was observed in 2020 due to self-isolation measures. However, post-pandemic, attacks surged beyond 2020 levels, with incidents involving minors increasing by 14.59% by 2023. This sparked public fear and prompted widespread dog poisoning incidents, as widely reported in the media. These challenges highlight the ongoing complexity of managing dog populations and mitigating associated risks in the Russian Federation.

The issue of dog bites in the Russian Federation has garnered considerable attention due to its significant public health and safety implications. A notable instance of this concern was reflected in the WHO Regional Office for Europe’s publication, Health Advice for Travelers to the 2018 FIFA World Cup in the Russian Federation [28]. This concern highlights the risk associated with rabies transmission, as the WHO estimates that dog-mediated rabies results in approximately 59,000 human deaths globally each year [29]. From 2012 to the present, the Russian Federation has reported 26 fatal cases of rabies. In 2022, two rabies-related deaths were documented, compared to six cases during the same period in 2021 [30]. A case of rabies in the attacking dog cannot be ruled out, as no diagnostic testing was performed prior to the animal being lethally dispatched by local police authorities.

### Age and vulnerability

The victim’s advanced age is a significant factor in this case. Existing literature [31–34] indicates that elderly individuals face a higher risk of severe outcomes in dog attacks due to factors such as reduced mobility, delayed defensive responses, and pre-existing health conditions [35–37], which may exacerbate the severity of injuries [7, 19]. Similarly, another fatal dog attack case report highlighted that older adults frequently sustain injuries in critical areas such as the neck. Recent literature provides examples, such as a case involving a 62-year-old woman who was fatally attacked by her own dog at her home [38], and another case involving a 61-year-old man who was attacked by two dogs while cycling in the countryside of southern Italy [39].

Males are significantly more likely to be bitten by dogs than females across all age groups, with studies reporting dog bite incidents in males occurring 1.4 to 3 times more frequently than in females [40–42]. However, one study noted that this trend does not apply to males over 60 years of age, who are bitten at similar rates to females within the same age group [43].

### Injury patterns

In this case, the injuries primarily consisted of bites to the neck and limbs, resulting from repeated, uncontrolled targeted biting, with the dog showing minimal responsiveness to the woman’s efforts to stop the attack. Consistent with previous literature, most injuries in adults occur on the extremities [40], while in children, over 70% of injuries involve the head, neck, and face [44]. The canine teeth of a carnivorous animal can produce “hole-and-tear” wounds [14], which, combined with superficial parallel lacerations and abrasions, are distinctive features of dog bites [45].

Fatal attacks to the neck region, including the carotid arteries and trachea, are consistent with attack patterns observed in fatal canine incidents [46]. The prolonged duration of the attack and significant bleeding from these wounds can contribute to the fatal outcome. Although morphometric analysis of canine teeth can assist in identifying the dog through the intercanine distance [47], severe cases involving avulsions and lacerations can make this identification impossible, as reported in this study. Additionally, dog bites can initially be mistaken for stab wounds [45]. A controversial case from France [48], initially suspected to be a homicide but later determined to be a fatal dog attack, revealed that the victim did not sustain injuries to the major cervical blood vessels. The neck wounds resulted in minimal bleeding, which did not require a transfusion or vascular suturing, although the victim experienced dissection of both carotid arteries.

According to De Munnynck and Van de Voorde (2002) [45], a forensic investigation of a fatal dog attack should encompass not only a thorough examination of the victim and the dog but also an assessment of the scene. They recommend a four-stage process: (1) gathering information on circumstances, witnesses, victim demographics, dog breed, and other relevant details; (2) examining the scene for footprints, environmental conditions, and body position; (3) collecting trace evidence, such as blood samples; and (4) documenting the scene with photographs, sketches, and diagrams. However, in our case, much of this traditional forensic work was circumvented [32] due to the availability of CCTV footage, which provided invaluable data for police investigators. To our knowledge, this is the first fatal dog attack to be documented in such a comprehensive manner using surveillance footage. While we did not have direct access to the footage or images, we reconstructed the sequence of events based on available transcripts.

### The critical role of CCTV footage in event reconstruction and behavioral assessment

Fatal dog attacks are more common in unwitnessed incidents and are often caused by exsanguination due to vascular trauma, air embolism from neck vein damage, or blunt craniofacial trauma resulting in skull fractures [49–52]. In similar cases [39, 53, 54], the absence of direct witnesses required forensic pathologists to determine which dogs were involved in the attack. In contrast, our case benefited significantly from CCTV footage, allowing for an accurate reconstruction of the sequence of events, including the initial confrontation, the duration and intensity of the attack, and the victim’s loss of consciousness. Beyond forensic analysis, the footage provided critical insights into stray dog behavior during an attack and the progressive nature of injuries sustained. This highlights the potential utility of surveillance footage in understanding fatal animal attacks, aiding both forensic investigations and public health interventions.

A PhD dissertation employing a mixed-methods research design explored the reliability of CCTV footage as forensic evidence [55], while another study [56] examined artefacts and distortions affecting the accuracy of such footage in forensic analysis and legal proceedings. As demonstrated in our case, CCTV footage serves as both an exclusionary tool and a valuable source of investigative information, significantly narrowing the scope of the forensic inquiry.

This case illustrates the critical role of CCTV surveillance in forensic investigations, not only by providing an objective account of the sequence of events but also by offering a rare opportunity to analyze prolonged aggression post-consciousness. The ability to document the sustained nature of the attack in real-time enhances our understanding of the behavioral patterns of aggressive dogs, reinforcing the need for enhanced public safety measures and effective stray animal management policies.

The fatal stray dog attack in November 2021 may be linked to seasonal and environmental stressors. November marks the shift to winter, often associated with reduced food availability, which can heighten aggression in stray dog populations. Although most dog bite incidents occur in warmer months [57–60], this case occurred during a period of high human activity (15:19:10) near a residential area. The dog’s behavior may have been influenced by competition for food and territorial stress. Additionally, the socio-environmental context of November 2021—when COVID-19 pandemic restrictions were easing, yet some measures such as non-working periods and partial lockdowns remained—likely exacerbated these risks.

The dog involved in the fatal attack was lethally dispatched by a local police officer shortly after the incident. While no forensic dental examination or bite mark analysis was performed to confirm the animal’s involvement, both eyewitness testimony (the female caretaker) and clear visual evidence from CCTV footage directly linked the specific dog to the attack. Although physical forensic correlation was absent, the convergence of eyewitness and video data provided a high degree of confidence in identification.

## Conclusion

This case highlights the intersection of human vulnerability, stray dog biting behavior, and the unique value of modern documentation methods in reconstructing fatal animal attacks. The availability of CCTV footage played a crucial role in accurately analyzing the sequence of events, offering forensic investigators an objective and detailed account of the attack. Beyond its significance in legal and forensic contexts, CCTV surveillance also serves as an essential public safety tool, helping authorities monitor, assess risk-prone areas, and implement timely interventions to prevent similar incidents.

Further research and intervention strategies are necessary to address the growing issue of stray dog aggression and to protect at-risk populations effectively. Strengthening efforts to manage stray dog populations—through improved control measures, responsible pet ownership programs, and stricter enforcement of public safety regulations—is essential in mitigating such threats.

## Key points

1. **Fatal stray dog attack** An elderly man in Greater Moscow region was fatally mauled, suffering severe bite injuries that led to exsanguination, highlighting the dangers of human-stray dog conflicts.

2. **CCTV Documentation** Surveillance footage played a crucial role in reconstructing the attack, offering objective evidence of the dog’s prolonged aggression and the victim’s inability to escape.

3. **Stray dog behavior** The attack exhibited sustained and repetitive biting behavior, aligning with patterns seen in fatal canine incidents, emphasizing the dangers posed by aggressive stray dogs.

4. **Public safety implications** This case highlights the need for comprehensive stray dog control measures, including sterilization programs, public awareness campaigns, and improved response strategies to prevent similar fatal encounters.

## Data Availability

Not applicable.
