# Correction: Fatal stray dog attack in Russian federation: a case report based on CCTV Documentation

**DOI:** 10.1007/s12024-025-01027-2

**Published:** 2025-05-30

**Authors:** Galina Zolotenkova, Rizky Merdietio Boedi, Oleg Viktorovich Lysenko, Nikolaos Angelakopoulos

**Affiliations:** 1 Department of Forensic Medicine, I.M. Sechenov First Moscow State Medical University, Moscow, Russian Federation; 2 Department of Dentistry, Faculty of Medicine, Universitas Diponegoro, Semarang, Indonesia; 3 Moscow Regional Research and Clinical Institute (MONIKI), Moscow, Russian Federation; 4 Department of Orthodontics and Dentofacial Orthopedics, University of Bern, Freiburgstrasse 7, Bern, 3010 Switzerland

**Correction: Forensic Science, Medicine and Pathology**

10.1007/s12024-025-01015-6

The paper “Fatal stray dog attack in Russian Federation: a case report based on CCTV documentation” has been published with incorrect author names sequence. Previously, the sequence is “Angelakopoulos Nikolaos, Merdietio Boedi Rizky, Lysenko Oleg Viktorovich, Zolotenkova Galina” while it should be “Galina Zolotenkova, Rizky Merdietio Boedi, Oleg Viktorovich Lysenko, Nikolaos Angelakopoulos”.

The original article has been corrected.

